# The Role of NFκB in Healthy and Preeclamptic Placenta: Trophoblasts in the Spotlight

**DOI:** 10.3390/ijms21051775

**Published:** 2020-03-05

**Authors:** Brooke Armistead, Leena Kadam, Sascha Drewlo, Hamid-Reza Kohan-Ghadr

**Affiliations:** 1 Department of Obstetrics, Gynecology and Reproductive Biology, Michigan State University, College of Human Medicine, Grand Rapids, MI 49503, USA; armiste9@msu.edu (B.A.); sdrewlo@msu.edu (S.D.); 2 Department of Obstetrics and Gynecology, Wayne State University School of Medicine, Detroit, MI 48202, USA; kadamleena1@gmail.com

**Keywords:** nuclear factor kappa-B, pregnancy, placenta, trophoblast, preeclampsia

## Abstract

The NFκB protein family regulates numerous pathways within the cell—including inflammation, hypoxia, angiogenesis and oxidative stress—all of which are implicated in placental development. The placenta is a critical organ that develops during pregnancy that primarily functions to supply and transport the nutrients required for fetal growth and development. Abnormal placental development can be observed in numerous disorders during pregnancy, including fetal growth restriction, miscarriage, and preeclampsia (PE). NFκB is highly expressed in the placentas of women with PE, however its contributions to the syndrome are not fully understood. In this review we discuss the molecular actions and related pathways of NFκB in the placenta and highlight areas of research that need attention

## 1. Introduction

An important family of proteins, known as nuclear-factor kappa-light chain of B cells (NFκB), regulate multiple pathways that impact cellular function. These include proliferation [1], differentiation [2], apoptosis [3], angiogenesis [4], epithelial to mesenchymal transition [5,6], and oxidative stress [7]. As well, NFκB is known for its role as a central mediator of inflammation and additionally for its roles modulating hypoxia-dependent gene expression [1,4,8], which are critically involved in placental development. 

The placenta is an important organ composed of trophoblast cells that arise from the extraembryonic layer of the blastocyst. The placenta conducts a range of functions that aim to support fetal growth and development, including temperature regulation, protection of the maternal micro-environment from infection, establishment of immunologic tolerance of the fetus, and to provide the exchange of gases, nutrients, and waste [9,10,11]. Proper placental development is essential for a successful pregnancy. In the first trimester of pregnancy, the placenta develops in a low oxygen environment [12]. NFκB is activated by the inflammation and hypoxia that occurs in early pregnancy. As well, NFκB can further upregulate genes in both of these pathways. Studies of murine pregnancies result in fetal demise by day 16 of gestation when NFκB proteins were deleted, which supports its role in pregnancy and placentation [2,13]. 

Abnormal placental development can lead to pregnancy complications, such as preeclampsia (PE). PE is a hypertensive disorder of pregnancy originated from the placenta, that affects up to 8% of all pregnancies [14]. It is the main cause of maternal and fetal morbidity and mortality worldwide [14]. PE is characterized by new onset of maternal hypertension occurring after 20 weeks of gestation, involving systemic endothelial dysfunction in the presence or absence of proteinuria [15]. There is no cure for PE and the primary method of treatment is placental and fetal delivery [10]. In severe cases, PE leads to preterm birth, which poses immediate and long-term health complications of the fetus and mother [14,16]. The exact etiology of PE is unknown however, as in severe cases it involves abnormal placental development and function [10,17,18,19]. 

During PE, the spiral arteries do not provide adequate amounts of nutrients and oxygen as the demand increases over pregnancy [20,21]. Placentas from women with PE develop in prolonged hypoxic state that extends beyond the physiological period in the first trimester [20,22]. Additionally, during PE, the placenta is exposed to excessive oxidative stress and inflammation, accompanied by abnormal trophoblast differentiation and increased secretion of anti-angiogenic proteins compared to healthy control patients [23,24,25]. Inflammation and oxidative stress conditions can increase NFκB activity and without coincidence, multiple reports show that women with PE exhibit up to 10-fold increase in NFκB expression in the placenta and maternal circulation compared to control pregnancies [26,27,28]. This suggests that NFκB may be implicated in PE pathophysiology. In this review, we discuss current knowledge on pathways regulated by NFκB that affect trophoblast differentiation and function during normal pregnancy and PE. We further highlight areas where more research would provide critical insights in placental physiology and disease.

## 2. The NFκB Family of Proteins

The NFκB protein family describes a group of proteins and their subunits that make up the Rel family [2]. These include c-Rel, Rel-A (p65), Rel-B, NFκB-1 (p50 and p105) and NFκB2 (p52 and p100) [1,7,13,29,30]. NFκB functions as a transcription factor that is expressed in nearly every mammalian cell [31]. It remains in its inactive form in the cytoplasm, as it is bound to inhibitor proteins, IκBs [1,2]. The cytoplasmic sequestering of NFκB ensures a rapid response to various stimuli [13]. 

### 2.1. NFκB Activation

NFκB activation begins by IκB phosphorylation and subsequent ubiquitination via IκB kinase complexes (IKKs, IKKα and IKKβ) to release NFκB [1]. Upon nuclear translocation, NFκB binds to κB DNA regulatory elements on the promoter of genes to modulate gene expression [1]. NFκB activation involves both canonical and non-canonical mechanisms for activation [7]. The canonical activation involves NFκB-1 and occurs by various ligand binding such as cytokine receptors, pattern recognition receptors, and tumor necrosis factor (TNF) super family receptors, including interleukin-1 (IL-1) receptors and toll like receptors (TLRs) [1,32]. Bacterial toxins such as lipopolysaccharide (LPS) activate NFκB-1 to initiate the production of pro-inflammatory cytokines [30,32]. Other endogenous molecules can activate NFκB-1 as well, such as damage-associated molecular patterns (DAMPs) [33]. DAMPs are secreted from necrotic or stressed cells and activate NFκB-1 through TLR receptor pathways to induce pro-inflammatory cascades [34]. The non-canonical activation of NFκB-2 can occur from ligand binding to lymphotoxin β receptor, B-cell activating factor receptor-3, and CD40 [7].

### 2.2. Actions of NFκB

NFκB can affect multiple pathways within the cell, including proliferation [1], differentiation [2], angiogenesis [4], hypoxia [4], epithelial to mesenchymal transition [5,6], and oxidative stress [7]. Probably one of the most known functions of NFκB is its role as a central mediator in inflammatory and immune response pathways [1]. NFκB initiates the production and secretion of pro- and anti-inflammatory cytokines and immune cell differentiation as well [35]. TNFα is a common downstream target of NFκB. It elicits pro-inflammatory cascades by also serving as a ligand to increase NFκB activity [36,37]. 

Hypoxia drives many gene expression pathways through the activation of NFκB, to increase perfusion and promote anaerobic metabolism to maintain a cellular energy balance [4]. Other proteins such as hypoxia inducible factors 1 and 2 (HIF1, HIF2) are major regulators of hypoxia-mediated gene expression pathways in the placenta [38]. Both HIF and NFκB proteins are activated through the oxygen sensing proteins: prolyl-hydroxylases (PHDs) and factor inhibiting HIFs (FIHs) [13]. During hypoxia, FIH can directly interact with IκBs to activate NFκB and PHD2 functions as a co-activator for Rel-A [13]. HIF1 controls both hypoxic and inflammatory pathways through NFκB activation to regulate cytokines and nitric oxide in the placenta [4]. 

NFκB function is often context dependent, as it is found to act as both a pro- and anti-oxidant mediator [7] and it similarly has pro- and anti-apoptotic functions [2]. Excessive NFκB activation is associated with oxidative stress and inflammation in numerous diseases, including atherosclerosis, osteoporosis, Alzheimer’s disease [28], inflammatory bowel disease (IBD), rheumatoid arthritis [1,32], and cancer [4]. NFκB is also shown to play a role in the pathogenesis of liver disease in pregnancy, known as intrahepatic cholestasis of pregnancy (ICP) [39]. As well, NFκB may contribute to pre-term birth through interactions with activator protein 1 (AP-1) to initiate the onset of labor [40].

Many inflammatory diseases exhibit high NFκB activity or find NFκB to be constitutively active and this poses harm to surrounding tissues, by sequestering immune cells and increasing production of pro-inflammatory molecules [41]. Many anti-inflammatory therapeutics target NFκB due to its gatekeeper role in eliciting inflammatory responses [1]. Targeting NFκB may be done through over expression of IkBs or inhibition of IKKs [41]. Multiple drugs that target inflammatory pathways impact NFκB activity, including aspirin and corticosteroids [41]. Other disease-modifying antirheumatic drugs (DMARDs) target NFκB indirectly, such as sulfasalazine, which is used to treat IBD to prevent NFκB activity in colon cells [31]. Anti-cytokine drugs are also used to treat Crohn’s disease and arthritis, including anakinra, an IL-1 receptor antagonist, and adalimamab, an anti-TNFα monoclonal antibody [31]. 

## 3. NFκB Actions in the Placenta during Normal Pregnancy and Preeclampsia

NFκB may be implicated in placental development in a number of ways. Mouse studies involving NFκB Rel-A knockouts and IKK-knockouts result in fetal demise at day 16 of pregnancy, showing that NFκB is essential in early development and placentation [2,13]. NFκB can exert protective roles through the activation of anti-apoptotic pathways during embryonic stress [2]. Furthermore, NFκB modulates several downstream pathways involved in trophoblast differentiation and function. Here, we discuss the involvement of NFκB during trophoblast differentiation in normal pregnancy and PE. 

### 3.1. NFκB throughout Normal Pregnancy

Inflammation is an important component to normal pregnancies and can be separated into three phases. While the first and third trimesters are considered to be pro-inflammatory, the second trimester is anti-inflammatory [42]. Prior to conception, NFκB expression is high in the decidua where it assists in regulating the implantation window [43]. The first trimester of pregnancy is considered to be pro-inflammatory due to the dominance of the pro-inflammatory cytokine profile identified from blood of pregnant women [42]. As embryo implantation occurs, it exerts an ‘open wound’ phenotype in the uterus causing secretion of pro-inflammatory cytokines [44]. 

Maintaining a physiological balance of pro- and anti-inflammatory cytokines is critical for a successful pregnancy. In a recent study, Kaislasuo et al. quantified the anti-inflammatory, interleukin-10 (IL-10), and pro-inflammatory, TNFα, cytokines in the blood serum of pregnant women. Their results show that IL-10 was significantly higher in women with normal pregnancies compared to women with pregnancy loss within 6–8 weeks of gestation [45]. Additionally, TNFα was significantly lower in women with normal pregnancy outcomes, compared to those with pregnancy loss at weeks 4–9 [45]. These results indicate that pregnancy loss may be associated with greater pro-inflammatory cytokines and further highlights the importance for proper balance and regulation of inflammation during early pregnancy [45]. The pro-inflammatory cytokine profile decreases towards the end of the first trimester and the second trimester exhibits an anti-inflammatory state as pregnancy progresses [42]. 

The third trimester of pregnancy is driven by a pro-inflammatory state [42]. NFκB is highly expressed in the decidua, where it assists in preparing for parturition by inducing cervical ripening and degradation of the extra-cellular matrix to initiate the rupture of placental membranes [43]. Moreover, NFκB is known to directly regulate factors that are present during labor, such as IL-8, cyclooxygenase-2 (COX2), and prostaglandins [43]. 

### 3.2. Placental Trophoblasts

The placenta is comprised of trophoblast cells which arise from the extra-embryonic trophectoderm layer of the blastocyst [12,46]. These cells develop into cytotrophoblasts (CTBs) and differentiate into two main lineages: the invasive extra-villous cytotrophoblasts (EVTs) and the villous cytotrophoblasts (VTs) [12]. Placental villi are formed at day 10 post-conception, and consist of two main villi types: a) floating villi which, at the start of the second trimester, are ‘bathed’ in maternal blood, and b) anchoring villi which secure placental attachment to the uterus in early pregnancy [9,46,47].

The base of the anchoring villi are composed of the human leukocyte antigen (HLA)-G+ proliferative column cytotrophoblasts (pCCTs) [9]. These cells differentiate into distal column cytotrophoblasts (dCCTs), as they migrate closer to the tip of the villi [12]. As these cells become detached from the column, they will differentiate to form interstitial or endovascular EVTs, which invade into the decidua and carry out their functions [12,46]. 

### 3.3. EVT Function and NFκB Actions in Normal Pregnancy

EVTs have critical functions during pregnancy. They facilitate maternal immune acceptance of the placenta and fetus, through their interactions with immune cells in the decidua, such as natural killer cells, macrophages, and T cells [11,48]. EVTs expand, differentiate, invade, and remodel maternal spiral arteries to permit low resistance high blood flow into the implantation site and support fetal growth throughout pregnancy [9]. During this process, the smooth muscle layer inside the arteries is completely removed and replaced by endovascular EVTs that acquire an endothelial-like phenotype [49], by a process known as pseudo-vasculogenesis. 

EVTs also function to regulate the oxygen conditions of the placenta. In early placental development, the EVTs will form a plug at the base of the maternal spiral arteries to prevent blood flow into the implantation site [50,51]. During this time, the placenta will develop in a low oxygen state [51]. The low oxygen helps to regulate the careful balance between CTB proliferation and VT and EVT differentiation [9,52,53]. The EVT plugs begin to dissolve around weeks 10–12 of gestation, which increases placental oxygen tension as the placenta becomes fully exposed to maternal blood [9,50,52,53,54]. 

Several studies suggest that NFκB may have a role in EVT function. EVT invasion is partially regulated by NFκB-induced secretion of cytokines, such as IL-6 and IL-8, from cells that act in an autocrine and paracrine manner [12,42,55]. Additionally, cytokines are secreted from the decidua to regulate EVT invasion [42].

Our group recently conducted a study using human first trimester EVT explants that were treated with LPS and observed increased EVT outgrowth compared to non-treated explants over a culture period of 24 hours at 3% O_2_ [56]. In addition, we treated the trophoblast-like cell line, HTR-8/SVneo with LPS and performed a matrigel invasion experiment. The LPS-treated cells showed a significant increase in the number of invaded cells compared to non-treated cells [56]. LPS treatment also increased expression of the invasion marker, integrin α1, and decreased expression of the non-invasive cell marker, integrin α6, compared to non-treated cells [56]. 

A major pathway that causes EVT invasion is epithelial to mesenchymal transition (EMT) [57] which is known to be regulated by NFκB [58,59]. During EMT, cells change their shape, adhesion molecules, and polarity to become invasive and drive cell migration [55]. This is facilitated by the production of matrix metalloproteases (MMPs) [60]. MMPs are secreted from the cell and degrade the extra-cellular matrix [60]. During low oxygen levels, as found in early pregnancy, NFκB upregulates the expression of MMP-2 and MMP-9 [58,59]. Both MMP-2 and -9 are known to have significant roles in EVT invasion. Liu et al. identified that invasion and migration of HTR-8/SVneo can be induced by the flavonoid, Baicalein, which activates NFκB and then upregulates MMP-9 expression [58]. 

Interestingly, NFκB regulates factors that both inhibit and stimulate invasion. In breast cancer cell lines, NFκB activation via TNFα was shown to promote EMT and invasion [61,62]. However, a study by Huber et al. showed opposite results using the HTR-8/SVneo cell line [63]. Treatment of HTR-8/SVneo with TNFα was shown to activate plasminogen activator inhibitor-1 (PAI-1) through NFκB, which impaired trophoblast invasion during a matrigel invasion assay [63]. Similar results were reported in a study by Bauer et al. in first trimester placental explants [64]. TNFα treatment increased PAI-1 expression, which inhibited EVT migration [64]. 

Tian et al. show that EMT occurs in primary small airway epithelial cells through TGF-β activation of NFκB [65]. However, secretion of TGF-β from the decidua is known to inhibit EVT invasion to provide protection of maternal tissues from over invasion [55]. These data suggest that NFκB may help coordinate EVT invasion to allow sufficient invasion, while simultaneously preventing over-invasion into maternal tissues. 

Besides stimulating invasion, the trophoblast-decidual crosstalk permits immunological acceptance of EVTs by the maternal tissues [9,11,55,66]. Cytokine secretion from the EVTs educates decidual immune cells by initiating differentiation or altering immune-cell response [2,48]. A study by Guzman-Genuino et al. recently demonstrated that trophoblasts can influence decidual B-cell differentiation in a co-culture system [67]. This immune education assists with trophoblast survival and maintenance of their migratory phenotypes during immunological insults such as infection [11,67]. 

### 3.4. EVT Function and NFκB Actions in Preeclampsia

The preeclamptic placenta is characterized by shallow EVT invasion into the decidua and reduced spiral artery remodeling, which prevents the vasculature expansion needed to permit low resistance high blood flow into the placental villi and implantation site [54,68]. Additionally, this causes intermittent placental perfusion that exposes the placenta to oxidative stress [69,70,71] and inflammation [28,72]. 

Several studies have reported that women with PE exhibit hyper-activation of NFκB, with its expression measured up to 10-fold in the placenta and maternal circulation, compared to control pregnancies [26,27,28]. The excessive activation of NFκB may increase pro-inflammatory cytokines and decrease regulatory and anti-inflammatory cytokines to promote an inflammatory state [73]. The inflammation and oxidative stress can also promote leukocyte activation and neutrophil infiltration in the endothelium of women with PE [28,72], contributing to the endothelial dysfunction. 

The changes in cytokine profile of women with PE may disrupt trophoblast-decidual crosstalk and EVT invasion. One example of this may be through NFκB activation of PAI-1. Multiple reports state that PAI-1 expression is significantly increased in the placenta of women with PE [74,75] and studies have shown PAI-1 can be induced via TNFα activation of NFκB, to decrease EVT invasion [63,64]. 

The increased inflammation may be explained by alterations in the toll-like receptor 4 (TLR4) signaling pathway that may enhance NFκB activation and is shown to play a role in PE [76]. Studies in animal models have shown that a low dose of inflammatory agents that target TLR4, such as LPS, is enough to upregulate placental expression of TLR4, which subsequently activates NFκB and causes PE-like symptoms [77]. Moreover, antagonizing the TLR4 signaling pathway was shown to block placental activation of NFκB and attenuate preeclampsia symptoms in murine models [78,79].

There appears to be an overlap of dysfunctional pathways that are present in the preeclamptic placenta which are in-part regulated by NFκB. However, there is a lack of critical studies investigating the direct impact of exacerbated activation of NFκB on EVT differentiation and function. This field of study requires more research due to the important functions of EVTs in healthy and abnormal pregnancies. 

### 3.5. VT Function and NFκB Actions in Normal Pregnancies

In the floating villi of the placenta, VTs differentiate as they fuse together forming the multi-nucleated fetal-maternal interface, known as the syncytium [68]. VT differentiation is regulated by important transcription factors, such as peroxisome proliferator activated receptor (PPAR)-γ [68] and glial cell missing 1 (GCM1) [80]. The primary function of the syncytium is to interact with maternal blood to provide nutrients, waste, and gas exchange between the mother and developing fetus [12,68].

VTs also function as the main source of placental protein and hormone production [9]. VTs produce and secrete pro- and anti-angiogenic proteins that assist in regulating placental and maternal angiogenesis, which are essential for generating a circulatory system that provides nutrients to the fetus [81,82]. Several proteins are important for angiogenic function, including the vascular endothelial growth factor (VEGF) proteins [48], fibroblast growth factor, and angiopoietins and their receptors [81]. NFκB may have a functional role in VTs, as it is known to modulate the expression of angiogenic proteins. 

Several cancer reports have shown a role for NFκB in coordinating angiogenesis by initiating gene expression of pro-angiogenic proteins: VEGF, IL-8, and MMP-9 [83,84,85,86,87]. Walton et al. identified direct regulation of VEGF-A by NFκB via a chromatin immunoprecipitation study in adult rat cardiomyocytes [88]. Included in the VEGF family of proteins is placental growth factor (PIGF), which is secreted by trophoblasts and is expressed under hypoxia and pro-inflammatory stimuli [52,85]. One of the primary functions of PIGF is to support angiogenesis and modulate trophoblast growth and differentiation [89]. NFκB has regulatory binding sites in the PIGF gene promoter and can initiate transcriptional activity of PIGF via Rel-A during hypoxia [85]. Endoglin (ENG) is another angiogenic protein that is highly expressed in the syncytium and is regulated by NFκB [90]. ENG functions as the cell surface receptor for TGF-β and assists in the production of vasodilatory gases, such as nitric oxide synthesis, to maintain vascular homeostasis [91]. 

NFκB may also have a role in regulating oxidative stress response pathways in VTs. Around 10–12 weeks of gestation, the placenta is exposed to maternal blood [49] which increases placental oxygen tension from 3% to 8% [92]. At this time, the VTs and the syncytium will exhibit an increase in oxidative stress as they adapt to the change in oxygen tension [92]. Reactive oxygen species (ROS) are generated during this process, including superoxide, hydroxide, and hydrogen peroxide (H_2_O_2_) [92]. NFκB is shown to be activated by the H_2_O_2_ oxidation process, which degrades IκB, allowing nuclear translocation and subsequent activation of NFκB [93]. NFκB may exert anti-apoptotic and antioxidant properties during this process by activating ROS sequestering molecules to decrease ROS during oxidative stress [7]. 

### 3.6. VT Function and NFκB Actions in Preeclampsia

As previously mentioned, placentas of women with preeclampsia are characterized by a prolonged hypoxic state with intermittent perfusion, which leads to a placenta exposed to high amounts of inflammation and oxidative stress. These conditions can suppress CTB proliferation and cause abnormal VT differentiation and defective syncytialization [10]. Abnormal differentiation and function of VT may also be responsible for the aberrant secretion of angiogenic proteins into the maternal-fetal circulation, which largely contributes to maternal vascular dysfunction, proteinuria, and hypertension [18,94]. 

In T cells, it has been shown that during oxidative stress, the cytosolic oxidation of H_2_O_2_ can activate NFκB [95]. When this occurs in excess, such as in PE, NFκB can directly upregulate downstream targets, like TNFα, to promote an inflammatory response [95]. This can also induce the production of placental-originated proteins that negatively affect vascular function. This includes endothelin-1 (ET-1) and soluble fms-like tyrosine kinase 1 (sFLT1) [96,97]. NFκB may upregulate anti-angiogenic factors that increase oxidative stressors like Arginase II in the maternal endothelium, causing additional damage and dysfunction of the vasculature in women with PE [98]. 

In a study investigating the effects of LPS on human first trimester explants, we observed a positive reaction of increased EVT outgrowth after LPS treatment [56]. However, LPS treatment of villous explants led to an increased secretion of pro-inflammatory cytokines IL-6, IL-1β, IL-8, RANTES, and TNFα, which induced trophoblast cell apoptosis [56]. Additionally, LPS significantly decreases GCM1 mRNA expression, which is a critical transcription factor driving VT differentiation in the villous explants [56]. 

Overall these studies suggest that NFκB expression is important in the placenta and the outcomes of NFκB activity largely depends on the context. It is unclear at what point NFκB expression may cause harm or when it is involved in the physiological events governing pregnancy. There is a clear lack of knowledge of the molecular mechanisms of NFκB regulation in VT differentiation and function in normal pregnancies, and PE and further studies are needed to close this gap. 

## 4. Conclusions

Throughout this review, we discussed the potential roles of NFκB during normal placental development and in the preeclamptic placenta. NFκB exerts its function during the prop-inflammatory and hypoxic state of the first trimester of normal pregnancies where it regulates production of the cytokines that promote EVT invasion and trophoblast-decidual crosstalk. NFκB is known to regulate angiogenic proteins, which happen to be secreted from VTs and have key roles in the development of a feto-placental vasculature system. Moreover, studies show that NFκB can exert anti-apoptotic and anti-oxidative properties, which is important during periods of oxidative stress which similarly occurs in VTs, such as upon placental exposure to maternal blood. 

**Figure 1:**
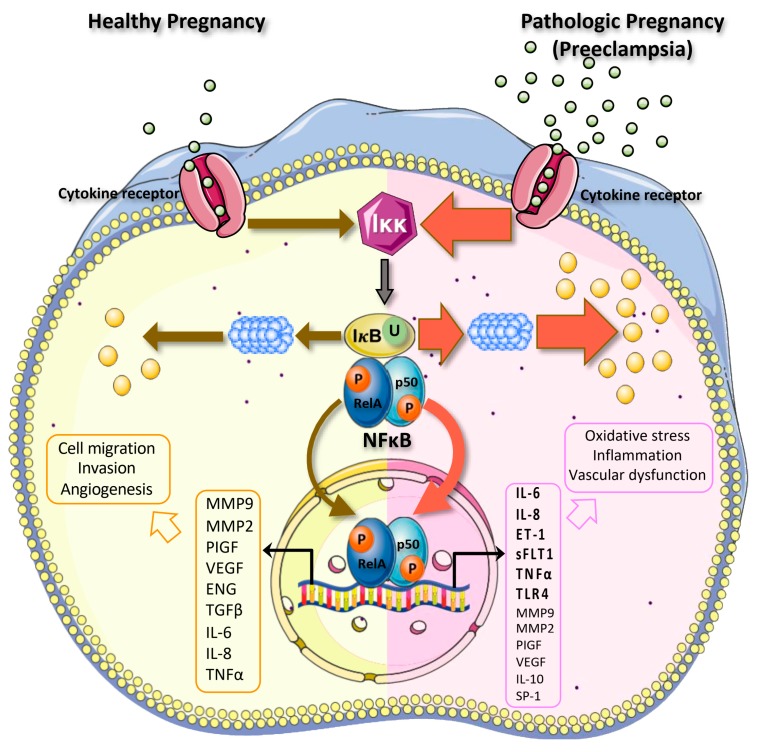
Actions of NFκB in the healthy and preeclamptic placenta. During normal pregnancies, NFκB functions to support the cellular migration, invasion, and production of angiogenic proteins from the placenta, through activation of several target genes. During pathological pregnancies such as preeclampsia (PE), NFκB is highly expressed as described by the red arrows. NFκB may induce expression of pro-inflammatory and anti-angiogenic proteins, further promoting oxidative stress, inflammation, and vascular dysfunction that occurs in PE.

Multiple pieces of evidence show that excessive activation of NFκB occurs in PE and this may exacerbate disease conditions [26,27,28]. In the preeclamptic placenta, EVT invasion is shallow, which prevents sufficient blood and oxygen from reaching the implantation site, resulting in intermittent oxygen perfusion and placental oxidative stress. Under these conditions, VTs exhibit abnormal differentiation, causing a defective syncytial layer, which exerts abnormal secretion of proteins into maternal-fetal circulation and causes maternal endothelial dysfunction. The effects of NFκB activity in the healthy and preeclamptic placenta are summarized in Figure 1. 

While NFκB is known to affect several pathways within the placenta that are also found to drive PE, it is still unclear how NFκB activation contributes to abnormal placental development and function. Future research on the roles of NFκB in the placenta during normal and pathological pregnancies would help to better understand human placentation, PE etiology, and identify possible therapeutic targets for PE.

## Abbreviations

NFκB	Nuclear-Factor Kappa-Light Chain of B Cells
PE	Preeclampsia
TNF	Tumor Necrosis Factor
IL-1	Interleukin-1
TLR	Toll-Like Receptor
LPS	Lipopolysaccharide
HIF1	Hypoxia Inducible Factor 1
HIF2	Hypoxia Inducible Factor 2
DAMPs	Damage-Associated Molecular Patterns
PHDs	Prolylhydroxylases
FIHs	Factor Inhibiting HIFs
IBD	Inflammatory Bowel Disease
ICP	Intrahepatic Cholestasis of Pregnancy
AP-1	Activator Protein-1
DMARDs	Disease-Modifying Antirheumatic Drugs
IL-10	Interleukin-10
COX2	Cyclooxygenase-2
CTB	Cytotrophoblast
EVT	Extravillous Cytotrophoblast
VT	Villous Cytotrophoblast
pCCT	Proliferative Column Cytotrophoblast
dCCT	Distal Column Cytotrophoblast
EMT	Epithelial-Mesenchymal Transition
PAI-1	Plasminogen Activator Inhibitor-1
TGFB	Transforming Growth Factor B
TLR4	Toll-Like Receptor 4
PPARG	Peroxisome Proliferator Activated Receptor G
GCM1	Glial Cell Missing 1
VEGF	Vascular Endothelial Growth Factor
PIGF	Placental Growth Factor
ENG	Endoglin
ROS	Reactive Oxygen Species
H_2_O_2_	Hydrogen Peroxide
ET-1	Endothelin-1
sFLT1	Soluble Fms-Like Tyrosine Kinase 1

## References

[1] Liu T., Zhang L.Y., Joo D., Sun S.C. (2017). NF-kappa B signaling in inflammation. Signal Transduct. Tar..

[2] Torchinsky A., Toder V. (2004). To die or not to die: The function of the transcription factor NF-kappa B in embryos exposed to stress. Am. J. Reprod. Immunol..

[3] Zhou A., Scoggin S., Gaynor R.B., Williams N.S. (2003). Identification of NF-kappa B-regulated genes induced by TNFalpha utilizing expression profiling and RNA interference. Oncogene.

[4] Cummins E.P., Comerford K.M., Scholz C., Bruning U., Taylor C.T. (2007). Hypoxic regulation of NF-kappa B signaling. Methods Enzymol..

[5] Huber M.A., Beug H., Wirth T. (2004). Epithelial-mesenchymal transition—NF-kappa B takes center stage. Cell Cycle.

[6] Huber M.A., Azoitei N., Baumann B., Grunert S., Sommer A., Pehamberger H., Kraut N., Beug H., Wirth T. (2004). NF-kappa B is essential for epithelial-mesenchymal transition and metastasis in a model of breast cancer progression. J. Clin. Invest..

[7] Lingappan K. (2018). NF-kappaB in Oxidative Stress. Curr. Opin. Toxicol..

[8] Taylor C.T. (2008). Interdependent roles for hypoxia inducible factor and nuclear factor-kappaB in hypoxic inflammation. J. Physiol..

[9] Knofler M., Haider S., Saleh L., Pollheimer J., Gamage T.K.J.B., James J. (2019). Human placenta and trophoblast development: Key molecular mechanisms and model systems. Cell. Mol. Life Sci..

[10] Lindheimer M.D., Roberts J.M., Cunningham F.G. (2009). Chesley’s Hypertensive Disorders in Pregnancy.

[11] Mor G., Kwon J.Y. (2015). Trophoblast-microbiome interaction: A new paradigm on immune regulation. Am. J. Obstet. Gynecol..

[12] Pollheimer J., Vondra S., Baltayeva J., Beristain A.G., Knofler M. (2018). Regulation of Placental Extravillous Trophoblasts by the Maternal Uterine Environment. Front. Immunol..

[13] D’Ignazio L., Rocha S. (2016). Hypoxia Induced NF-kappaB. Cells.

[14] Jeyabalan A. (2013). Epidemiology of preeclampsia: Impact of obesity. Nutr. Rev..

[15] (2019). ACOG Practice Bulletin No. 202: Gestational Hypertension and Preeclampsia. Obstet. Gynecol..

[16] McCarthy F.P., Drewlo S., English F.A., Kingdom J., Johns E.J., Kenny L.C., Walsh S.K. (2011). Evidence Implicating Peroxisome Proliferator-Activated Receptor-gamma in the Pathogenesis of Preeclampsia. Hypertension.

[17] Phipps E.A., Thadhani R., Benzing T., Karumanchi S.A. (2019). Pre-eclampsia: Pathogenesis, novel diagnostics and therapies. Nat. Rev. Nephrol..

[18] O’Brien M., Baczyk D., Kingdom J. (2017). Soluable factor versus microparticle—mediated endothelial cell dysfunction in severe pre-eclampsia. Bjog.-Int. J. Obstet. Gy..

[19] Rana S., Lemoine E., Granger J.P., Karumanchi S.A. (2019). Preeclampsia: Pathophysiology, Challenges, and Perspectives. Circ Res.

[20] Fisher S.J. (2015). Why is placentation abnormal in preeclampsia?. Am. J. Obstet. Gynecol..

[21] Cartwright J.E., Fraser R., Leslie K., Wallace A.E., James J.L. (2010). Remodelling at the maternal-fetal interface: Relevance to human pregnancy disorders. Reproduction.

[22] Lyall F., Robson S.C., Bulmer J.N. (2013). Spiral artery remodeling and trophoblast invasion in preeclampsia and fetal growth restriction: Relationship to clinical outcome. Hypertension.

[23] Aouache R., Biquard L., Vaiman D., Miralles F. (2018). Oxidative Stress in Preeclampsia and Placental Diseases. Int. J. Mol. Sci..

[24] Sanchez-Aranguren L.C., Prada C.E., Riano-Medina C.E., Lopez M. (2014). Endothelial dysfunction and preeclampsia: Role of oxidative stress. Front. Physiol..

[25] Chiarello D.I., Abad C., Rojas D., Toledo F., Vazquez C.M., Mate A., Sobrevia L., Marin R. (2020). Oxidative stress: Normal pregnancy versus preeclampsia. Biochim. Biophys. Acta Mol. Basis Dis..

[26] Silva Carmona A., Mendieta Zeron H. (2016). NF-kappaBeta and SOD expression in preeclamptic placentas. Turk. J. Med. Sci..

[27] Zhao L., Wang H., Zhang W., Tian X., Sun Q. (2017). Serum NF-kappaBp65, TLR4 as Biomarker for Diagnosis of Preeclampsia. Open Med..

[28] Vaughan J.E., Walsh S.W. (2012). Activation of NF-kappaB in placentas of women with preeclampsia. Hypertens Pregnancy.

[29] Wakeland A.K., Soncin F., Moretto-Zita M., Chang C.W., Horii M., Pizzo D., Nelson K.K., Laurent L.C., Parast M.M. (2017). Hypoxia Directs Human Extravillous Trophoblast Differentiation in a Hypoxia-Inducible Factor-Dependent Manner. Am. J. Pathol..

[30] Schulze-Luehrmann J., Ghosh S. (2006). Antigen-receptor signaling to nuclear factor kappa B. Immunity.

[31] Herrington F.D., Carmody R.J., Goodyear C.S. (2016). Modulation of NF-kappaB Signaling as a Therapeutic Target in Autoimmunity. J. Biomol. Screen..

[32] Lawrence T. (2009). The nuclear factor NF-kappaB pathway in inflammation. Cold Spring Harb. Perspect Biol..

[33] Roh J.S., Sohn D.H. (2018). Damage-Associated Molecular Patterns in Inflammatory Diseases. Immune. Netw..

[34] Patel S. (2018). Danger-Associated Molecular Patterns (DAMPs): The Derivatives and Triggers of Inflammation. Curr. Allergy Asthma Rep..

[35] Dorrington M.G., Fraser I.D.C. (2019). NF-kappaB Signaling in Macrophages: Dynamics, Crosstalk, and Signal Integration. Front. Immunol..

[36] Hayden M.S., Ghosh S. (2014). Regulation of NF-kappaB by TNF family cytokines. Semin. Immunol..

[37] Schutze S., Wiegmann K., Machleidt T., Kronke M. (1995). TNF-induced activation of NF-kappa B. Immunobiology.

[38] Ietta F., Wu Y., Winter J., Xu J., Wang J., Post M., Caniggia I. (2006). Dynamic HIF1A regulation during human placental development. Biol. Reprod..

[39] Zhang Y., Hu L., Cui Y., Qi Z., Huang X., Cai L., Zhang T., Yin Y., Lu Z., Xiang J. (2014). Roles of PPARgamma/NF-kappaB signaling pathway in the pathogenesis of intrahepatic cholestasis of pregnancy. PLoS ONE.

[40] Peng Q., Liu Y., Dong M., Xu F., Huang J., Chen J., Li X., Zhang J., Zhang W. (2018). Interaction between NF-kappaB and AP-1 and their intracellular localization at labor in human late pregnant myometrial cells in vivo and in vitro. Medicine.

[41] Verma I.M. (2004). Nuclear factor (NF)-kappaB proteins: Therapeutic targets. Ann. Rheum. Dis..

[42] Mor G. (2008). Inflammation and pregnancy—The role of toll-like receptors in trophoblast-immune interaction. Ann. Ny. Acad. Sci..

[43] Sakowicz A. (2018). The role of NFB in the three stages of pregnancy—Implantation, maintenance, and labour: A review article. Bjog. Int. J. Obstet. Gy..

[44] Mor G., Cardenas I., Abrahams V., Guller S. (2011). Inflammation and pregnancy: The role of the immune system at the implantation site. Ann. NY Acad. Sci..

[45] Kaislasuo J., Simpson S., Petersen J.F., Peng G., Aldo P., Lokkegaard E., Paidas M., Pal L., Guller S., Mor G. (2019). IL-10 to TNFalpha ratios throughout early first trimester can discriminate healthy pregnancies from pregnancy losses. Am. J. Reprod. Immunol..

[46] Turco M.Y., Moffett A. (2019). Development of the human placenta. Development.

[47] Gude N.M., Roberts C.T., Kalionis B., King R.G. (2004). Growth and function of the normal human placenta. Thromb. Res..

[48] Tilburgs T., Crespo A.C., van der Zwan A., Rybalov B., Raj T., Stranger B., Gardner L., Moffett A., Strominger J.L. (2015). Human HLA-G+ extravillous trophoblasts: Immune-activating cells that interact with decidual leukocytes. Proc. Natl. Acad. Sci. USA.

[49] Zhou Y., Genbacev O., Damsky C.H., Fisher S.J. (1998). Oxygen regulates human cytotrophoblast differentiation and invasion: Implications for endovascular invasion in normal pregnancy and in pre-eclampsia. J. Reprod. Immunol..

[50] Chang C.W., Wakeland A.K., Parast M.M. (2018). Trophoblast lineage specification, differentiation and their regulation by oxygen tension. J. Endocrinol..

[51] James J.L., Stone P.R., Chamley L.W. (2006). The regulation of trophoblast differentiation by oxygen in the first trimester of pregnancy. Hum. Reprod. Update.

[52] Fujita D., Tanabe A., Sekijima T., Soen H., Narahara K., Yamashita Y., Terai Y., Kamegai H., Ohmichi M. (2010). Role of extracellular signal-regulated kinase and AKT cascades in regulating hypoxia-induced angiogenic factors produced by a trophoblast-derived cell line. J. Endocrinol..

[53] Kilburn B.A., Wang J., Duniec-Dmuchowski Z.M., Leach R.E., Romero R., Armant D.R. (2000). Extracellular matrix composition and hypoxia regulate the expression of HLA-G and integrins in a human trophoblast cell line. Biol. Reprod..

[54] Roberts J.M., Escudero C. (2012). The placenta in preeclampsia. Pregnancy Hypertens.

[55] Davies J.E., Pollheimer J., Yong H.E.J., Kokkinos M.I., Kalionis B., Knofler M., Murthi P. (2016). Epithelial-mesenchymal transition during extravillous trophoblast differentiation. Cell Adhes. Migr..

[56] Kadam L., Kilburn B., Baczyk D., Kohan-Ghadr H.R., Kingdom J., Drewlo S. (2019). Rosiglitazone blocks first trimester in-vitro placental injury caused by NF-kappaB-mediated inflammation. Sci. Rep..

[57] DaSilva-Arnold S.C., Zamudio S., Al-Khan A., Alvarez-Perez J., Mannion C., Koenig C., Luke D., Perez A.M., Petroff M., Alvarez M. (2018). Human trophoblast epithelial-mesenchymal transition in abnormally invasive placenta. Biol. Reprod..

[58] Liu J., Lv S.S., Fu Z.Y., Hou L.L. (2018). Baicalein Enhances Migration and Invasion of Extravillous Trophoblasts via Activation of the NF-kappaB Pathway. Med. Sci. Monit..

[59] Tabruyn S.P., Griffioen A.W. (2008). NF-kappa B: A new player in angiostatic therapy. Angiogenesis.

[60] Hiden U., Eyth C.P., Majali-Martinez A., Desoye G., Tam-Amersdorfer C., Huppertz B., Ghaffari Tabrizi-Wizsy N. (2018). Expression of matrix metalloproteinase 12 is highly specific for non-proliferating invasive trophoblasts in the first trimester and temporally regulated by oxygen-dependent mechanisms including HIF-1A. Histochem. Cell Biol..

[61] Pires B.R., Mencalha A.L., Ferreira G.M., de Souza W.F., Morgado-Diaz J.A., Maia A.M., Correa S., Abdelhay E.S. (2017). NF-kappaB Is Involved in the Regulation of EMT Genes in Breast Cancer Cells. PLoS ONE.

[62] Dong R., Wang Q., He X.L., Chu Y.K., Lu J.G., Ma Q.J. (2007). Role of nuclear factor kappa B and reactive oxygen species in the tumor necrosis factor-alpha-induced epithelial-mesenchymal transition of MCF-7 cells. Braz. J. Med. Biol. Res..

[63] Huber A.V., Saleh L., Bauer S., Husslein P., Knofler M. (2006). TNFalpha-mediated induction of PAI-1 restricts invasion of HTR-8/SVneo trophoblast cells. Placenta.

[64] Bauer S., Pollheimer J., Hartmann J., Husslein P., Aplin J.D., Knofler M. (2004). Tumor necrosis factor-alpha inhibits trophoblast migration through elevation of plasminogen activator inhibitor-1 in first-trimester villous explant cultures. J. Clin. Endocrinol. Metab..

[65] Tian B., Widen S.G., Yang J., Wood T.G., Kudlicki A., Zhao Y., Brasier A.R. (2018). The NFkappaB subunit RELA is a master transcriptional regulator of the committed epithelial-mesenchymal transition in airway epithelial cells. J. Biol. Chem..

[66] Coates M.S., Alton E.W.F.W., Brookes D.W., Ito K., Davies J.C. (2016). Increased Respiratory Syncytial Virus Burden Leads to More Rapid Cell Death in Phe508del Bronchial Epithelial Cells. Thorax.

[67] Guzman-Genuino R.M., Dimova T., You Y., Aldo P., Hayball J.D., Mor G., Diener K.R. (2019). Trophoblasts promote induction of a regulatory phenotype in B cells that can protect against detrimental T cell-mediated inflammation. Am. J. Reprod. Immunol..

[68] Kadam L., Kohan-Ghadr H.R., Drewlo S. (2015). The balancing act—PPAR-gamma’s roles at the maternal-fetal interface. Syst. Biol. Reprod. Med..

[69] Hladunewich M., Karumanchi S.A., Lafayette R. (2007). Pathophysiology of the clinical manifestations of preeclampsia. Clin. J. Am. Soc. Nephrol..

[70] Regnault T.R., Galan H.L., Parker T.A., Anthony R.V. (2002). Placental development in normal and compromised pregnancies—A review. Placenta.

[71] Soleymanlou N., Jurisica I., Nevo O., Ietta F., Zhang X., Zamudio S., Post M., Caniggia I. (2005). Molecular evidence of placental hypoxia in preeclampsia. J. Clin. Endocrinol. Metab..

[72] Shah T.J., Walsh S.W. (2007). Activation of NF-kappaB and expression of COX-2 in association with neutrophil infiltration in systemic vascular tissue of women with preeclampsia. Am. J. Obstet. Gynecol..

[73] Harmon A.C., Cornelius D.C., Amaral L.M., Faulkner J.L., Cunningham M.W., Wallace K., LaMarca B. (2016). The role of inflammation in the pathology of preeclampsia. Clin. Sci..

[74] Ye Y., Vattai A., Zhang X., Zhu J., Thaler C.J., Mahner S., Jeschke U., von Schonfeldt V. (2017). Role of Plasminogen Activator Inhibitor Type 1 in Pathologies of Female Reproductive Diseases. Int. J. Mol. Sci..

[75] Fabbro D., D’Elia A.V., Spizzo R., Driul L., Barillari G., Di Loreto C., Marchesoni D., Damante G. (2003). Association between plasminogen activator inhibitor 1 gene polymorphisms and preeclampsia. Gynecol. Obstet. Invest..

[76] Koga K., Mor G. (2010). Toll-like receptors at the maternal-fetal interface in normal pregnancy and pregnancy disorders. Am. J. Reprod. Immunol..

[77] Xue P., Zheng M., Gong P., Lin C., Zhou J., Li Y., Shen L., Diao Z., Yan G., Sun H. (2015). Single administration of ultra-low-dose lipopolysaccharide in rat early pregnancy induces TLR4 activation in the placenta contributing to preeclampsia. PLoS ONE.

[78] Liu Y., Yang J., Bao J., Li X., Ye A., Zhang G., Liu H. (2017). Activation of the cholinergic anti-inflammatory pathway by nicotine ameliorates lipopolysaccharide-induced preeclampsia-like symptoms in pregnant rats. Placenta.

[79] Gong P., Liu M., Hong G., Li Y., Xue P., Zheng M., Wu M., Shen L., Yang M., Diao Z. (2016). Curcumin improves LPS-induced preeclampsia-like phenotype in rat by inhibiting the TLR4 signaling pathway. Placenta.

[80] Baczyk D., Drewlo S., Proctor L., Dunk C., Lye S., Kingdom J. (2009). Glial cell missing-1 transcription factor is required for the differentiation of the human trophoblast. Cell Death Differ..

[81] Reynolds L.P., Redmer D.A. (2001). Angiogenesis in the placenta. Biol. Reprod..

[82] Geva E., Ginzinger D.G., Zaloudek C.J., Moore D.H., Byrne A., Jaffe R.B. (2002). Human placental vascular development: Vasculogenic and angiogenic (branching and nonbranching) transformation is regulated by vascular endothelial growth factor-A, angiopoietin-1, and angiopoietin-2. J. Clin. Endocrinol. Metab..

[83] Ko H.M., Seo K.H., Han S.J., Ahn K.Y., Choi I.H., Koh G.Y., Lee H.K., Ra M.S., Im S.Y. (2002). Nuclear factor kappaB dependency of platelet-activating factor-induced angiogenesis. Cancer Res..

[84] Kofler S., Nickel T., Weis M. (2005). Role of cytokines in cardiovascular diseases: A focus on endothelial responses to inflammation. Clin. Sci..

[85] Cramer M., Nagy I., Murphy B.J., Gassmann M., Hottiger M.O., Georgiev O., Schaffner W. (2005). NF-kappaB contributes to transcription of placenta growth factor and interacts with metal responsive transcription factor-1 in hypoxic human cells. Biol. Chem..

[86] Huang S., Pettaway C.A., Uehara H., Bucana C.D., Fidler I.J. (2001). Blockade of NF-kappaB activity in human prostate cancer cells is associated with suppression of angiogenesis, invasion, and metastasis. Oncogene.

[87] Tong Q., Zheng L., Lin L., Li B., Wang D., Huang C., Li D. (2006). VEGF is upregulated by hypoxia-induced mitogenic factor via the PI-3K/Akt-NF-kappaB signaling pathway. Respir. Res..

[88] Walton C.B., Matter M.L. (2015). Chromatin Immunoprecipitation Assay: Examining the Interaction of NFkB with the VEGF Promoter. Methods Mol. Biol..

[89] De Falco S. (2012). The discovery of placenta growth factor and its biological activity. Exp. Mol. Med..

[90] Kapur N.K., Morine K.J., Letarte M. (2013). Endoglin: A critical mediator of cardiovascular health. Vasc. Health Risk Manag..

[91] Gregory A.L., Xu G., Sotov V., Letarte M. (2014). Review: The enigmatic role of endoglin in the placenta. Placenta.

[92] Pereira R.D., De Long N.E., Wang R.C., Yazdi F.T., Holloway A.C., Raha S. (2015). Angiogenesis in the placenta: The role of reactive oxygen species signaling. Biomed. Res. Int..

[93] Schreck R., Rieber P., Baeuerle P.A. (1991). Reactive oxygen intermediates as apparently widely used messengers in the activation of the NF-kappa B transcription factor and HIV-1. EMBO J..

[94] Rajakumar A., Powers R.W., Hubel C.A., Shibata E., von Versen-Hoynck F., Plymire D., Jeyabalan A. (2009). Novel Soluble Flt-1 Isoforms in Plasma and Cultured Placental Explants from Normotensive Pregnant and Preeclamptic Women. Placenta.

[95] Hirota K., Murata M., Sachi Y., Nakamura H., Takeuchi J., Mori K., Yodoi J. (1999). Distinct roles of thioredoxin in the cytoplasm and in the nucleus. A two-step mechanism of redox regulation of transcription factor NF-kappaB. J. Biol. Chem..

[96] Parrish M.R., Murphy S.R., Rutland S., Wallace K., Wenzel K., Wallukat G., Keiser S., Ray L.F., Dechend R., Martin J.N. (2010). The effect of immune factors, tumor necrosis factor-alpha, and agonistic autoantibodies to the angiotensin II type I receptor on soluble fms-like tyrosine-1 and soluble endoglin production in response to hypertension during pregnancy. Am. J. Hypertens..

[97] Eddy A., Chapman H., Brown D.T., George E. (2020). Differential Regulation of sFlt-1 Splicing by U2AF65 and JMJD6 in Placental-Derived and Endothelial Cells. Biosci. Rep..

[98] Sankaralingam S., Xu H., Davidge S.T. (2010). Arginase contributes to endothelial cell oxidative stress in response to plasma from women with preeclampsia. Cardiovasc. Res..

